# Early Rupture of Iatrogenic Cystic Artery Pseudoaneurysm After Unsuccessful Laparoscopic Cholecystectomy: A Case Report

**DOI:** 10.7759/cureus.22865

**Published:** 2022-03-05

**Authors:** Prokopis Christodoulou, Stavros-Chrysovalantis Liapis

**Affiliations:** 1 Surgical Department, General Hospital of Volos, Volos, GRC

**Keywords:** pseudoaneurysm of cystic artery, selective artery embolization, laparoscopic complications, laparoscopic cholecystectomy, pseudoaneurysm

## Abstract

Laparoscopic cholecystectomy has been established as the gold-standard method to deal with symptomatic cholelithiasis and cholecystitis. Although, like any other surgical procedure, it may have complications that affect the mortality and morbidity of patients. More specifically, the cystic artery pseudoaneurysm is considered a rare complication of laparoscopic cholecystectomy, which despite its rarity, may be fatal for the patient. Herein, we present the case of a 67-year-old man with a ruptured iatrogenic cystic artery pseudoaneurysm in the early postoperative period after laparoscopic cholecystectomy that converted to open wherein a cholecystostomy catheter was placed. The patient was hospitalized in our surgical unit, and he was treated with cystic artery embolization initially and secondary with elective open cholecystectomy.

## Introduction

Cystic artery pseudoaneurysm (CAP) is a rare complication that may occur after laparoscopic cholecystectomy and the incidence of it is uncertain, but if we consider the overall vascular injuries of hepatic arteries and their branches, the presence of aneurysms or pseudoaneurysms is estimated at 2% [1]. Rupture of it can occur either in the early or late postoperative period, and in most cases, 50 days may pass from the surgery until the onset of rupture [2]. CAPs are most associated with laparoscopic cholecystectomy, but they can also occur as a result of cholelithiasis, acute or chronic cholecystitis, or as an idiopathic condition [2, 3]. According to Taghavi et al. in a review of the literature, the most common cause of pseudoaneurysms was laparoscopic cholecystectomy [2]. In this case, we present the rupture of an iatrogenic CAP in the early postoperative period after an unsuccessful laparoscopic cholecystectomy, which was managed through elective vascular embolization.

## Case presentation

A 67-year-old male underwent laparoscopic cholecystectomy, which was converted to open cholecystectomy, wherein a cholecystostomy catheter was finally placed as Calot’s elements were difficult to identify due to chronic inflammation and fibrosis, in another hospital. On the 5th postoperative day, he was presented to our emergency department as he noticed blood flow from the cholecystostomy catheter. The patient had only one previous surgery for an inguinal hernia three years ago from his medical history. Upon arrival, his vital signs were: blood pressure 100/72 mmHg, heart rate 98 bpm, SpO2 98%, temperature 36.8 degrees Celsius. Clinical examination was performed and revealed, upon inspection of blood output from the catheter, mild tenderness at palpation of the right hypochondriac region, and normal bowel sounds. Initially, blood examinations were performed, and intravenous crystalloid fluids were administered. Table 1 shows the patient's blood results upon arrival.

**Table 1:** Patient's blood results upon arrival. HCT: hematocrit; HGB: hemoglobin; PLT: platelets; CRP: C-reactive protein; Cr: blood creatinine; BUN: blood urea nitrogen; TBIL: total bilirubin; AST: aspartate transaminase; ALT: alanine transaminase

Blood analysis upon arrival	Patient's results	Reference values
HCT	28%	35%-47%
HGB	7.9 gr/dL	11.5-15.5 gr/dL
PLT	320.0 K/μL	150.0-400.0 K/μL
CRP	74 mg/L	up to 5 mg/L
Cr	0.8 mg/dL	0.9-1.4 mg/dL
BUN	45 mg/dL	10-55 mg/dL
TBIL	1.10 mg/dL	up to 1.2 mg/dL
AST	52 IU/L	up to 40 IU/L
ALT	43 IU/L	up to 40 IU/L

**Figure 1:**
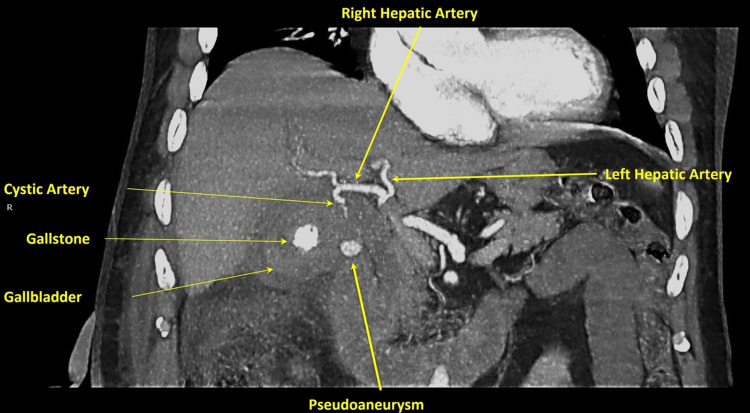
Coronal CT angiography reconstruction indicates a pseudoaneurysm arising from the cystic artery.

**Figure 2:**
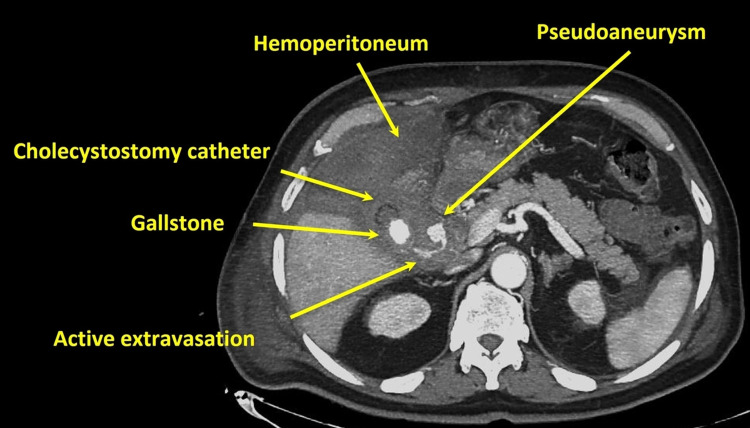
Axial CT angiography reconstruction indicates active extravasation from cystic artery pseudoaneurysm and hemoperitoneum in perihepatic space.

The patient underwent an abdominal CT scan with an intravenous contrast agent and reconstruction of it into CT angiography which revealed rupture of a cystic artery pseudoaneurysm with active extravasation and hemoperitoneum in the perihepatic space (Figures 1, 2).

**Figure 3:**
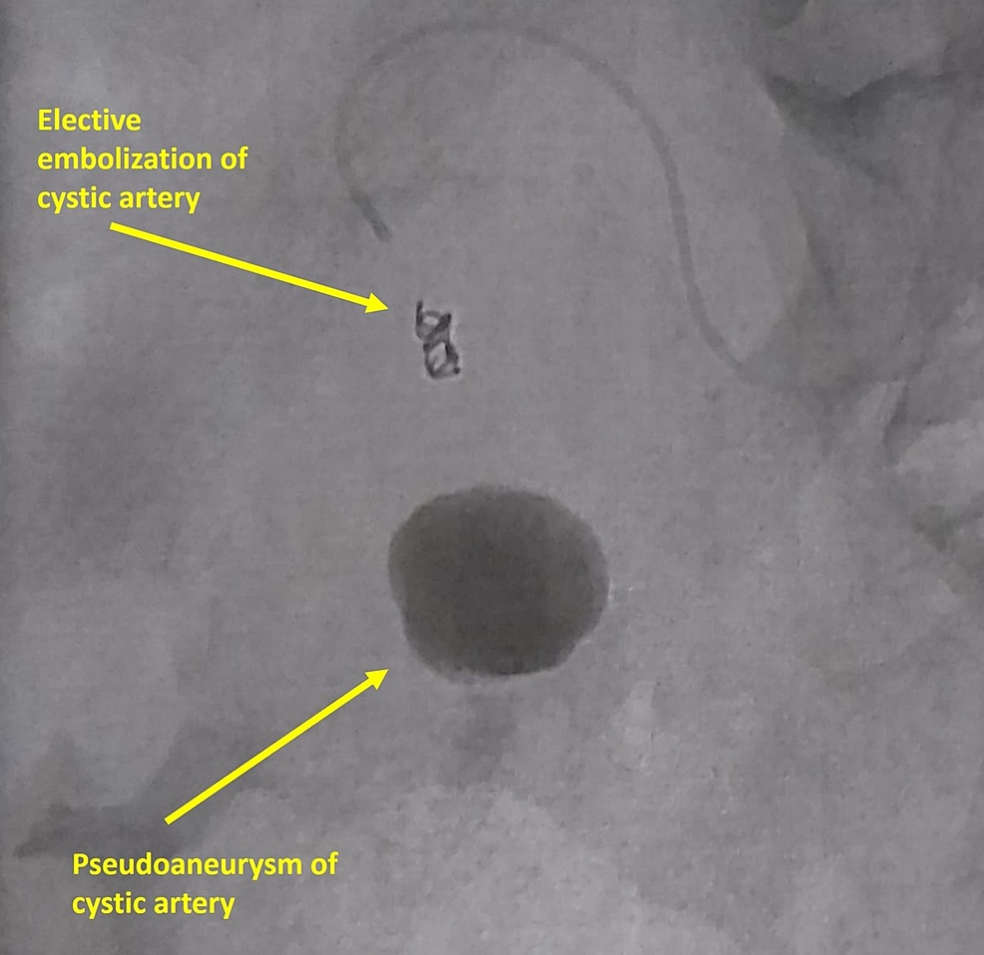
Image during embolization.

**Figure 4:**
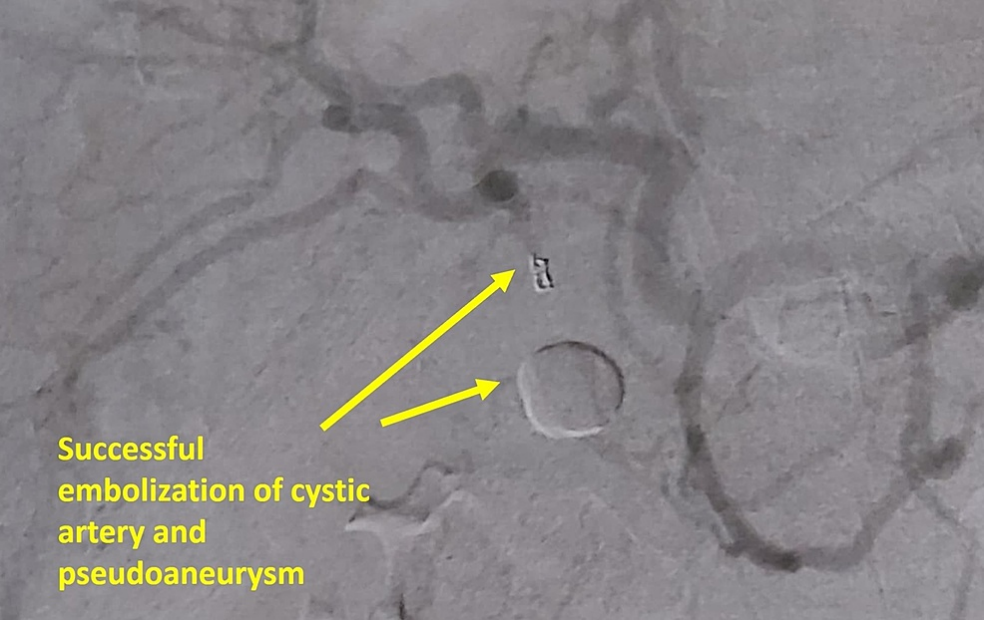
The result of successful embolization.

Then, the patient underwent an urgent angioembolization. Elective embolization of the cystic artery with coil was performed successfully (Figures 3, 4). Eventually, the patient was hospitalized in our surgical unit after embolization for four days under close monitoring and intravenous liquids. On the second day of hospitalization due to decreased level of hemoglobin (6.9 g/dl), the patient received one unit of red blood cells (RBCs). On all the days of hospitalization, the patient was stable without any blood output from the cholecystostomy catheter. On the fourth day of hospitalization, he was discharged, clinically improved and hemodynamically stable, with good tolerance of oral feeding and normal defecation. The patient came back after one week for a scheduled cholecystectomy. We performed an open cholecystectomy, and the patient was discharged on the second postoperative day without any adverse events.

## Discussion

Vascular injuries in laparoscopic cholecystectomy although rare, are an existing clinical entity that may be harmful to the patient. Vascular injuries may occur after the trocar insertion or during the dissection of the cystic artery [4]. Another described pathogenetic mechanism is the inflammation theory, which affects the vessel’s wall and leads to wall debilitation and eventually to the formation of pseudoaneurysm which can explain the association between acute or chronic cholecystitis and the presence of CAP [5]. In a large multi-center study, the incidence of vascular injuries in 77,604 patients that underwent laparoscopic cholecystectomy was 0.29%, which corresponded to 193 patients [6]. In 73 patients out of 193, the vascular injury occurred in the cystic artery, which was the most affected vessel [6]. Parthenis et al. in a review of literature mentioned the rarity of vascular pseudoaneurysms after laparoscopic surgeries and identified 66 relative cases where only three referred to cystic artery pseudoaneurysm and most of them concerned the right hepatic artery [7]. Another more recent review by Lampropoulos et al. confirmed that the right hepatic artery was the most common site of pseudoaneurysm development, followed by the cystic artery [8]. Among laparoscopic procedures, laparoscopic cholecystectomy is associated more often with the formation of pseudoaneurysm followed by laparoscopic nephrectomies [7]. Clinical presentation of CAP may be in the early or late postoperative period, although in most cases, may occur in the first postoperative month [9]. Its clinical manifestations include haemobilia, rupture with hemoperitoneum, hemorrhagic shock, and the Quincke’s triad, which is characterized by jaundice, abdominal pain, and gastrointestinal bleeding [9]. In this case, our patient presented since he noticed blood flow from the cholecystostomy catheter without having signs of hypovolemic shock, but the abdominal CT scan revealed hemoperitoneum in the perihepatic area with active extravasation from the CAP, so we assume that if the patient's arrival was delayed there would be signs of shock. Regarding the diagnosis of this rare clinical entity, it requires high clinical suspicion, especially in patients that are presented in the early postoperative period after laparoscopic cholecystectomy with abdominal pain, gastrointestinal bleeding, or signs of hypovolemic shock [8]. The most reliable radiological examination to diagnose CAPs is a triple-contrast abdominal CT scan or a CT angiography, as they can reveal even small CAPs [8]. More specifically, CT angiography is considered the method of choice to detect pseudoaneurysms [8]. Pseudoaneurysms even if they are asymptomatic need to be treated as there is a high risk of complications that may be fatal for the patient [10]. The risk of rupture varies within a range of 21%-80%, which increases the overall mortality of patients up to 40% [10]. The options that we have available to treat pseudoaneurysms and more specific CAPs are vascular embolization of the cystic artery, which is a minor invasive method, and laparotomy with ligation or excision of pseudoaneurysm [10]. Vascular embolization is preferable as it reduces mortality and morbidity at a rate of 25% in comparison with laparotomy [10]. If the vascular embolization fails, then it is obvious that the patient must undergo surgical intervention [10]. After successful vascular embolization of CAPs, the definite treatment includes elective open cholecystectomy [11]. Still, there is no agreement and no guidelines regarding the time of elective surgery and if this is going to be performed directly after embolization or later. In the literature, in one case, the patient underwent open cholecystectomy the day after embolization, while in another case the surgery was performed 10 days later [11, 12]. Tapnio et al., in a case series regarding three patients with CAPs and successful embolization of each one, performed cholecystectomy at different time-lapses, as in one patient performed after one day from the embolization, in another patient after two months, and the third patient underwent percutaneous cholecystostomy prior to embolization [13]. The placement of a cholecystostomy catheter due to difficult identification of Calot's elements highlights the importance of a non-aggressive approach as complications can arise, such as vascular injuries or injury of the common bile duct. Percutaneous cholecystostomy and open cholecystostomy are favorable procedures and contribute to the prevention of complications, especially in patients with severe inflammation and comorbidities [14, 15]. Both methods can be used as bridging methods until the definitive treatment with laparoscopic or open cholecystectomy [14, 15].

## Conclusions

Cystic artery pseudoaneurysm is a rare complication after laparoscopic cholecystectomies that may be fatal for the patient, and more specifically in the case of rupture. We highlight the necessity of high clinical suspicion to diagnose this disease, especially in patients with signs of hypovolemic shock short after laparoscopic cholecystectomy. It is important to underline the role of angioembolization to manage these vascular complications as it has lower adverse events than emergency laparotomy. Furthermore, in cholecystectomies where the identification of the cystic artery and cystic duct is difficult, the placement of a cholecystostomy catheter can prevent complications like vascular injuries or injury of the common bile duct. We believe that it will be useful to develop guidelines regarding this disease to not misdiagnose patients even in the asymptomatic phase, place them under a surveillance program, and treat high-risk patients at the right time.
